# Effect of synthesis methods on the activity of NiO/Co_3_O_4_ as an electrode material for supercapacitor: in the light of X-ray diffraction study†

**DOI:** 10.1039/d3ra05200a

**Published:** 2024-01-02

**Authors:** Amtul Nashim, Soumyashree Pany, Kulamani Parida

**Affiliations:** a Centre for Nano Science and Nano Technology, Institute of Technical Education and Research, Siksha ‘O’ Anusandhan (Deemed to be University) Bhubaneswar 751019 India paridakulamani@yahoo.com kulamaniparida@soa.ac.in

## Abstract

The formation of heterostructures by combining individual components (NiO and Co_3_O_4_) is a preferred approach to enhance electrochemical performance as it leads to improved charge transfer and surface reaction kinetics. In the present work, a NiO/Co_3_O_4_ composite was prepared by two methods. First, neat NiO and Co_3_O_4_ were prepared by adopting the hydrothermal method followed by the formation of the composite (i) by a hydrothermal route (NC-Hydro) and (ii) by a calcination route (NC-Cal). NC-Hydro composite shows a specific capacity of 176 C g^−1^ at 1 A g^−1^ of current density in the three-electrode system in a 2 M KOH solution as an electrolyte with 90% cyclic retention after 5000 cycles at 4 A g^−1^. NC-Cal shows a specific capacity of 111 C g^−1^ at 1 A g^−1^ with 75% cyclic retention. The coulombic efficiency of NC-Hydro was 86.3% while for NC-Cal it was 42.3%. The reason behind the superior electrochemical performance of NC-Hydro in comparison to NC-Cal may be the large interlayer spacing and lattice parameters of the former, which provide large space for redox reactions. The unit cell volume of the composites was more than that of the constituents. This study reveals that the composites prepared by the hydrothermal method have superior electrochemical properties in comparison to composites prepared by the calcination method.

## Abstract 2

The composite prepared by the hydrothermal method presented superior electrochemical properties compared to the composite prepared by calcination.

## Graphical abstract

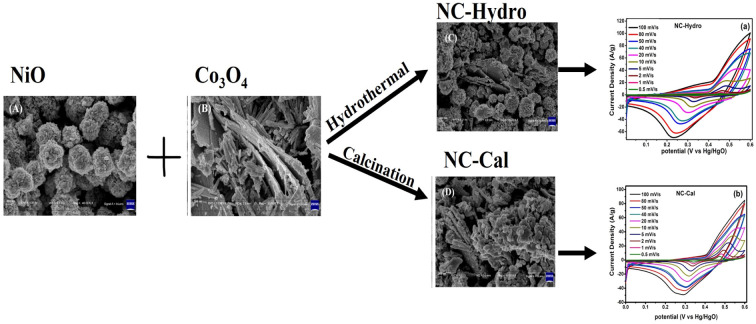

## 1. Introduction

To utilize a sustainable form of energy, besides generating clean and green energy, its storage is an essential part as well. In general, supercapacitors are considered for the storage of electric energy in a clean and green way.^1^ In terms of energy storage, supercapacitors offer advantages over traditional capacitors and batteries as they have superior capacitance, high power density, and long-term stability. Rechargeable batteries and supercapacitors can play an important role in future portable electronic devices and zero-emission electric vehicles in the future.^2^ In general, electrode materials for supercapacitors can be divided into three types according to charge storage mechanism: (i) capacitive materials in which the surface charges are accumulated electrostatically, (ii) pseudocapacitive materials in which rapid reversible faradaic reaction occurs near the electrode surface, and (iii) battery-type materials in which deep surface faradaic reactions occur.^1–5^ The selection of electrode materials for supercapacitors is the foremost step to achieve high specific capacitance along with optimal energy density, power density, and cyclic stability. The transition metal oxides/hydroxides, such as MnO_2_, Co_3_O_4_, MoO_3_, NiO, RuO_2_, *etc.* are found to be active materials for pseudocapacitors as they possess high redox activities, high theoretical capacities, high reversibility, *etc.*^6,7^ Among these, RuO_2_ possesses high theoretical specific capacitance (1400–2000 F g^−1^) and, therefore, it is extensively studied by researchers but its high production costs limit its practical application in the field.^8,9^ Moreover, single-component transition metal oxides, such as MnO_2_, SnO_2_, NiO, ZnO, and Co_3_O_4_, are used as a substitute for RuO_2_ owing to properties, such as non-toxic, natural abundance, environment-friendliness, and high specific capacity.^10–14^ Regardless, they suffer from low electronic conductivity, cyclic stability, and energy density, *etc.* It is well known that in comparison to single components, heterostructures that have been formed by combining single components are more favorable for electrochemical performance due to the improvement in charge transfer and surface reaction kinetics.^15,16^ Among all single-component metal oxides, NiO and Co_3_O_4_ have extensively been studied as electrode materials for electrochemical applications because of their affordability, environment-friendliness, corrosion stability in alkaline solution, high redox activity, and high theoretical capacity (NiO ∼2584 F g^−1^; Co_3_O_4_ ∼3560 F g^−1^).^17,18^ However, they have drawbacks, including low electronic conductivity, unsatisfactory life cycle, and low specific capacitance, which hinder their practical applications. However, when NiO and Co_3_O_4_ are combined to form a heterostructure, the productive effect of both can be seen and their individual drawbacks can be suppressed to some extent.^19–24^

In this work, we synthesized NiO/Co_3_O_4_ by adopting a facile and green method. Two methods were used for the synthesis here by keeping a fixed ratio between NiO and Co_3_O_4_: (i) hydrothermal method for synthesis of neat metal oxides followed by using the same hydrothermal method to prepare the composite, and (ii) hydrothermal method for neat metal oxides formation followed by thermal annealing process for composite formation. The composite prepared by these methods has been investigated for usage as the anode in a supercapacitor. The present work correlates the activity of supercapacitors with XRD analysis. The findings are discussed in detail in their respective sections. This study reveals that the composite prepared by the hydrothermal method presented superior electrochemical properties in comparison to the composite prepared by the calcination method.

## 2. Experimental section

### 2.1 Synthesis process

#### 2.1.1. Synthesis of NiO

0.38 g of Ni(CH_3_CO_2_)_2_·4 H_2_O and 0.72 g of urea were mixed in 80 mL of water and stirred for 30 minutes. The whole solution was then transferred to a Teflon-lined steel autoclave followed by heating at 150 °C for 5 h in a programmable electric oven without stirring to perform the hydrothermal reaction. After cooling naturally to room temperature, the sample was washed with water several times followed by ethanol washing. The sample was then dried at 80 °C for 12 h. The dried sample was collected and subjected to calcination at 300 °C for 2 h at a heating rate of 10 °C per minute.

#### 2.1.2. Synthesis of Co_3_O_4_

1.8 g of Co(NO_3_)_2_·6H_2_O and 1.08 g of urea were mixed in 80 mL of water and stirred for 30 minutes. The solution was then transferred to a Teflon-lined steel autoclave and the hydrothermal process was conducted at 110 °C for 16 h in a programmable electric oven without stirring. After cooling, the sample was washed with water and ethanol several times and dried at 80 °C for 12 h. Finally, the sample was calcined at 450 °C for 2 h at a heating rate of 10 °C per minute.

#### 2.1.3. Synthesis of NiO/Co_3_O_4_ (hydrothermal)

For the synthesis of NiO/Co_3_O_4_ composite, NiO and Co_3_O_4_ were mixed in a 2 : 1 ratio in 50 mL water and stirred for 15 minutes, followed by the addition of 50 mL of water. The solution was then transferred to a Teflon-lined steel autoclave for the hydrothermal process at 160 °C for 16 h in a programmable electric oven without stirring. After cooling, the sample was washed with water and ethanol several times and dried at 80 °C for 12 h. The dried sample of NiO/Co_3_O_4_ thus obtained has been abbreviated as NC-Hydro.

#### 2.1.4. Synthesis of NiO/Co_3_O_4_ (calcination)

**Scheme 1:**
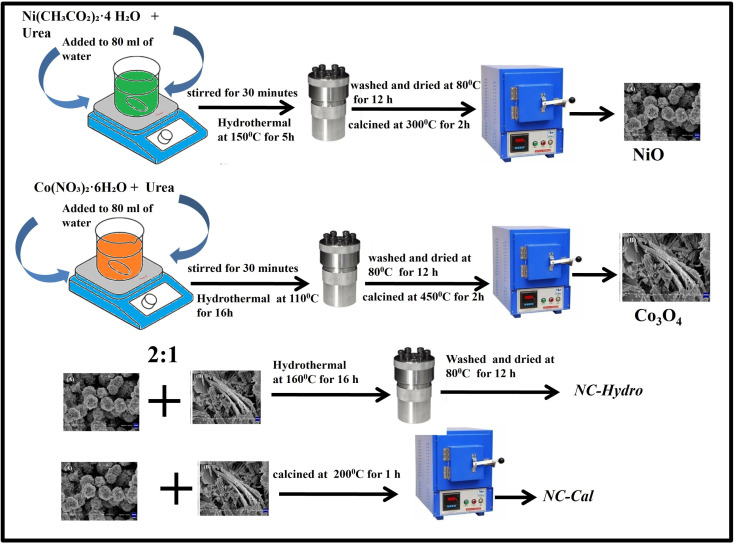
Schematic representation of the synthesis of NC-Hydro and NC-Cal.

For the synthesis of NiO/Co_3_O_4_ composite by the calcination method, the same amount of NiO and Co_3_O_4_ (as taken in hydrothermal synthesis) were taken in 60 mL of water and sonicated for 60 minutes, followed by stirring at 250 rpm for 18 h. The sample was then collected and calcined at 200 °C for 1 h at a heating rate of 5^°^/min. The as-synthesized sample was abbreviated as NC-Cal. Scheme 1 represents the synthesis of neat and composites.

### 2.2 Characterization

The prepared samples were characterized by X-ray diffraction (XRD), X-ray photoelectron spectroscopy (XPS), and field-emission scanning electron microscopy (FESEM). Rigaku-Ultima IV powder diffractometer was used to record the XRD pattern of the samples using a CuKα radiation source (*λ* = 0.154 nm) within the 2*θ* range of 10 to 80^°^. LaB_6_ NIST standard was used to calibrate the instrumental broadening of diffraction peaks. XPS measurement was performed on a Thermo scientific ESCA LABXi^+^ with a non-monochromatic X-ray source. Binding energy correction was performed using the C 1s peak of 284.6 eV as a reference. FESEM images were collected on a Carl Zeiss Model Supra 55, Germany. Electrochemical measurements were performed with a potentiostat/galvanostat (IVIUM-VERTEX).

### 2.3 Fabrication of the electrode

The electrochemical measurements were carried out by using three electrode systems in a 2 M aqueous solution of KOH as an electrolyte. The sample deposited on Ni foam was taken as working electrode, Pt wire as counter electrode and Hg/HgO as reference electrode with working potential range of 0–0.6 V. Cyclic voltammetry, galvanostatic charge–discharge, and electrochemical kinetics were studied in detail. Before use, nickel foam (area of 1.5 × 1 cm^2^) was washed with HCl, water, and acetone for 10 minutes in a sonicator, respectively.^20,21^ Nickel foam was then dried at 80 °C for 12 h in a vacuum oven. The drop-casting method was used to deposit the sample over the nickel foam (the area of deposition was 1 × 1 cm^2^). The ratio of 80 : 16 : 4 (sample : carbon black : Nafion, *i.e.*, 4 mg : 0.8 mg : 4 μL) was maintained for deposition onto the nickel foam. After deposition, the electrode (nickel foam with sample deposited on it) was dried at 80 °C for 12 h in a vacuum oven. Before using the electrode, 10 MPa pressure was applied to the foam for 10 seconds. The mass deposited on the nickel foam was found to be 2.94 mg.

## 3. Results and discussions

### 3.1 X-ray diffraction (XRD) analysis

**Fig. 1:**
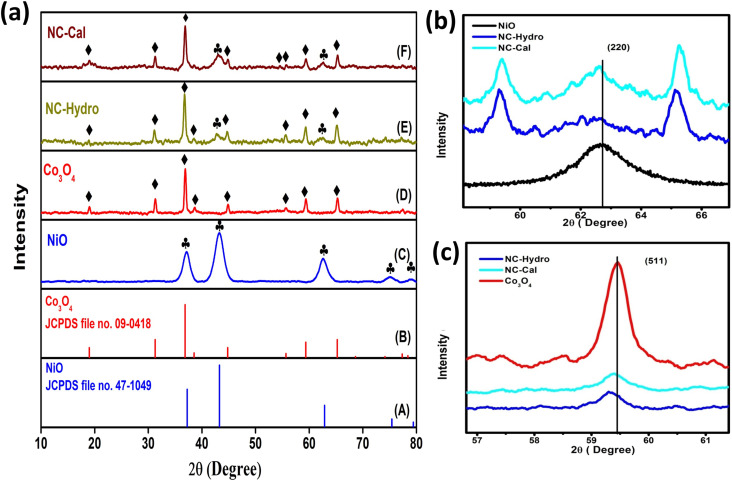
(a) The XRD patterns in the XRD diffractogram of (C) NiO, (D) Co_3_O_4_, (E) NC-Hydro, and (F) NC-Cal, in comparison to those from standard powder diffraction of (A) NiO (JCPDS no-47-1049) and (B) Co_3_O_4_ (JCPDS no- 09-0418). Fig. 1(b) and (c) shows the shifting of XRD peaks towards lower 2*θ* values along the lattice planes, (220) and (511), respectively. The symbol 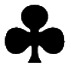 indicates the peaks of NiO and ♦ is used to denote the peaks of Co_3_O_4_.

Fig. 1(a) illustrates the XRD patterns in the XRD diffractogram of NiO, Co_3_O_4_, NC-Hydro, and NC-Cal, respectively. The diffraction peaks of NiO are found at 2*θ* values of 37.2, 43.3, 62.7, 75.4, and 79.3 and correspond to the planes, (111), (200), (220), (311), and (222), respectively. These values are well matched with the cubic NiO phase (JCPDS file no. 47-1049; Fig. 1(A)).^25^ Similarly, for Co_3_O_4_, the diffraction peak is found around 2*θ* values of 19, 31.3, 36.8, 38.5, 44.8, 55.7, 59.4, and 65.2, respectively, with the corresponding planes, (111), (220), (311), (222), (400), (422), (511), and (440) that are matched with the cubic Co_3_O_4_ phase (JCPDS file no. 09-0418; Fig. 1 (B)). Fig. 1(E) and (F) correspond to the diffraction peaks of NC-Hydro and NC-Cal, respectively.^26^ In Fig. 1(E) and (F), the diffraction peaks of both NiO and Co_3_O_4_ can be seen in composite NC-Hydro and NC-Cal indicating the formation of NiO/Co_3_O_4_ composite by both the hydrothermal and calcination methods. The crystallite size (*D*_*hkl*_) and micro-strain (*ε*) of the composites were calculated by using eqn (1) and (2),^27,28^ where *D*_*hkl*_ is the crystallite size that is normal to the reflection plane (*hkl*) in nm; *λ* is the wavelength of X-ray used in nm; *θ* is the Bragg angle; *β*_*hkl*_ is the width of the diffraction beam used (rad), which is calculated after correcting the instrumental broadening (*β*_0_ = 0.08 rad); and *K* is the shape factor of the crystallite size (*K* = 0.9, when *β*_*hkl*_ is defined as the half-high width of diffraction peaks)1
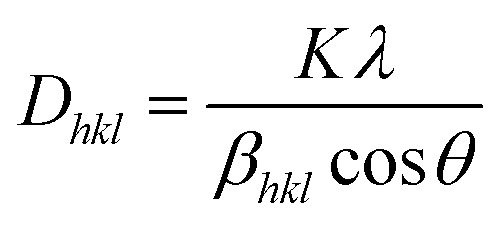
2
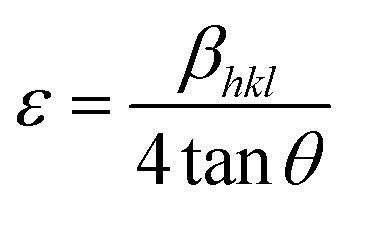


The interlayer spacing (*d*) is calculated using Bragg's eqn (3). Where *λ* is the wavelength of the X-ray used; *n* is the order of diffraction; and *θ* is the angle of diffraction.^29,30^

3 *nλ* = 2*d* sin *θ*

The degree of crystallinity is calculated by using eqn (4), where *X*_c_ is the degree of crystallinity; *β*_*hkl*_ is the full width at half maximum of (*hkl*) the reflection after correcting the instrumental broadening (*β*_0_ = 0.08 rad); and *K*_A_ is the constant set of 0.24.^31^4
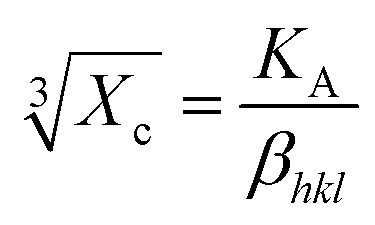


The lattice parameters for the cubic structure (*a* = *b* = *c*) are calculated using eqn (5); here, *h*, *k*, and *l* are the Miller indices, and *d* is the interlayer spacing.^30^5
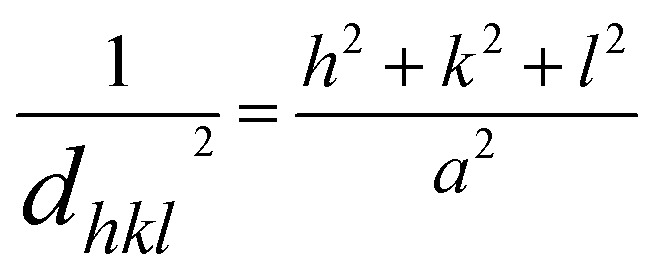


**Fig. 2:**
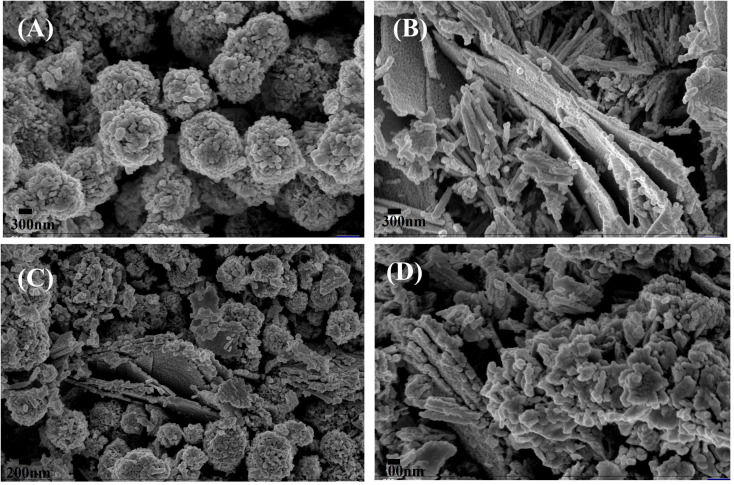
The FESEM micrographs of (A) NiO, (B) Co_3_O_4_, (C) NC-Hydro, and (D) NC-Cal.

Table 1 depicts the average crystallite size, average micro-strain, interlayer spacing, lattice parameters, cell volume, and crystallinity of the materials along the lattice planes, (220) and (511), respectively, for NiO and Co_3_O_4_. The crystallite size for the NiO phase upon forming a composite with Co_3_O_4_ is found to be 5.1 and 6.67 nm, respectively, for NC-Hydro and NC-Cal. Similarly, the average crystallite size of Co_3_O_4_ for NC-Hydro and NC-Cal, respectively, is found to be 23.93 and 24.9 nm. The average crystallite size of both the phases, *i.e.*, NiO and Co_3_O_4_, is found to be lower for NC-Hydro as compared to NC-Cal, proving the importance of hydrothermal techniques. The micro-strain of the NC-Cal is more than that of NC-Hydro; this may be because the calcination method causes greater defect and strain than the materials prepared by hydrothermal methods. Therefore, micro-strain, in the case of NC-Cal, is higher. While comparing the interlayer spacing of the materials along the lattice planes, (220) and (511), lattice expansion was observed in both the cases of NC-Hydro and NC-Cal. Fig. 1(b) represents the (220) lattice plane of NiO, NC-Hydro, and NC-Cal. Fig. 2(a) shows the shifting of XRD peaks towards lower 2*θ* values along the lattice plane (220) with respect to NiO in the case of composite materials. It is well known that whenever XRD peaks shift towards a lower 2*θ* value, lattice expansion occurs; consequently, a larger space is made available for redox reactions, resulting in increasing electrochemical properties.^32^ The same decrease in trend was observed when the XRD peak was considered along the lattice plane (511) as shown in Fig. 1(c), *i.e.*, lattice expansion in composites occurred in comparison to Co_3_O_4_. However, the interlayer spacing of NC-Hydro was more expanded in comparison to NC-Cal, which may be one of the reasons behind its stable electrochemical properties.^32^ The degree of crystallinity (*X*_c_) decreases after the formation of the composite as depicted in Table 1. This means that after the formation of the composite, its amorphous nature increases, which is a good sign of the enhancement of electrochemical properties. Here, the *X*_c_ is comparable for both NC-Hydro and NC-Cal. It is known that the higher the micro-strain, the higher will be the amorphous nature. An amorphous nature with a small crystallite size is beneficial for electrochemical activity.^33^ The lattice parameters of the composites increase with respect to the neat constituents. Consequently, the unit cell volume shows expansion which may facilitate the intercalation and deintercalation of the electrolytes during the electrochemical process.

**Table 1:** The average crystallite size, micro-strain, interlayer spacing, and crystallinity of the NiO, Co_3_O_4_, NC-Hydro, and NC-Cal

Sample	Average crystallite size (nm)		Average micro-strain (1 × 10^−2^ lines per m^2^)	Interlayer spacing, *d* (in nm)		Lattice parameters (*a* = *b* = *c*, cubic system) (Å)		Unit cell volume V (Å)^3^		Crystallinity	
				(220)	(511)	(220)	(511)	(220)	(511)	(220)	(511)
NiO	5.3		1.5 × 10^−2^	1.4802	—	4.186	—	73.34	—	0.0023	—
Co_3_O_4_	30.79		0.3 × 10^−2^	—	1.5524	—	8.064	—	524.4	—	0.322
NC-Hydro	NiO	5.1	1.1 × 10^−2^	1.4830	1.5562	4.194	8.085	73.77	528.49	0.0014	0.18
	Co_3_O_4_	23.9									
NC-Cal	NiO	6.6	1.8 × 10^−2^	1.4824	1.5549	4.192	8.075	73.65	526.53	0.0019	0.15
	Co_3_O_4_	24.9									

### 3.2 Field-emission scanning electron microscopy (FESEM)

Fig. 2 shows the FESEM images of NiO, Co_3_O_4_, NC-Hydro, and NC-Cal, respectively. Fig. 2(A) presents the FESEM image of NiO, where NiO particles assemble to form a brain-like structure. Fig. 2(B) presents the FESEM image of Co_3_O_4_, where it can be observed that the Co_3_O_4_ particles aggregate to form rod-like structures that are again assembled to form 2D sheet-like structures. Both rod and 2D sheet-like structures are present in Co_3_O_4_. 2D sheet-like structures play a vital role in increasing the electrochemical stability, flexibility, and tunable properties of an electrode.^34^Fig. 2(C) and (D) show the FESEM images of NC-Hydro and NC-Cal respectively. FESEM images of NC-Hydro and NC-Cal are presented at various magnitudes in Fig. S1.† By observing the FESEM images of both NC-Hydro and NC-Cal, it was found that NiO/Co_3_O_4_ composites prepared by hydrothermal and calcination method shared the same type of images, *i.e.*, both consist of brain-like structures, along with rods and sheet-like structure. This indicates that the stable structure of NiO and Co_3_O_4_, even after hydrothermal and calcination treatments, retains their original structures.

### 3.3 X-ray photoelectron spectroscopy (XPS)

**Fig. 3:**
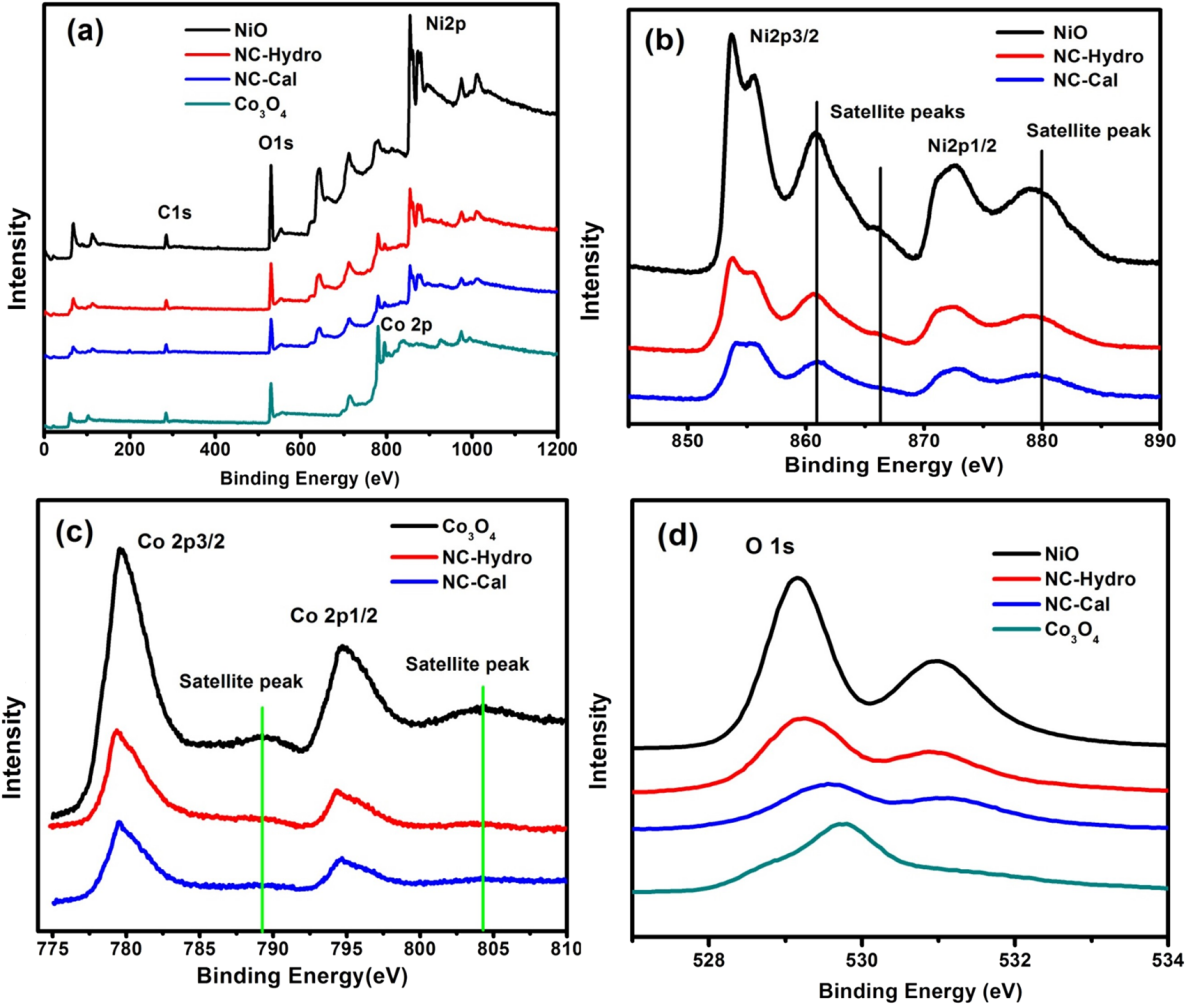
The XPS spectra of NiO, NC-Hydro, NC-Cal, and Co_3_O_4_, (a) the survey scan spectra (b) Ni 2p, (c) Co 2p, and (d) O 1s.

XPS is used to detect the chemical states of the materials. Fig. 3(a) shows the XPS survey scan spectra of NiO, NC-Hydro, NC-Cal, and Co_3_O_4_, respectively, indicating the presence of Ni, O, and Co. Fig. 3(b)–(d) represents the XPS spectra of NiO, NC-Hydro, NC-Cal, and Co_3_O_4_ for Ni 2p, Co 2p, and O 1s, respectively. From Fig. 3(b) and (c), the shifting of the XPS peaks of Ni and Co from their respective neat NiO and Co_3_O_4_ peaks can be easily seen; this may be due to the interaction between NiO and Co_3_O_4_ in the composites. Fig. S2(a)–(j)† represents the deconvoluted peaks of Ni, Co, and O, respectively, for NiO, NC-Hydro, and NC-Cal. The XPS peaks of Ni in the NiO, NC-Hydro, and NC-Cal were split into two spin–orbit components, *i.e.*, Ni 2p3/2 (at ∼853.5 eV) and Ni 2p1/2 (at ∼872.3 eV). The XPS peaks for Ni 2p3/2 found in the range of 853.5 to 853.8 eV represent Ni^2+^, and the peaks at ∼855 eV are due to Ni^3+^ with two satellite peaks at ∼860.8 and 865.6 eV, respectively. The peak for Ni 2p1/2 is found around 872 eV, along with a satellite peak at ∼879 eV. The presence of Ni^2+^ and Ni^3+^ states confirms the existence of non-stoichiometric NiO.^35^ Similarly, the XPS peaks of Co for Co_3_O_4_, NC-Hydro, and NC-Cal are split into two spin–orbit components, *i.e.*, Co2p3/2 (∼779 eV) and Co2p1/2 (∼795 eV). The XPS peaks at ∼779 eV and 780 eV for Co 2p3/2 represent Co^3+^ and Co^2+^ states, respectively, along with a satellite peak at ∼786.25 eV. The XPS peak at ∼795 eV is assigned to the Co 2p1/2 for the Co^3+^ oxidation state with a satellite peak at ∼803 eV. The difference between the spin–orbit splitting energy states of Co, *i.e.*, Co 2p3/2 and Co 2p1/2, was ∼15 eV, indicating the existence of Co^2+^ and Co^3+^; which is in good agreement with our XPS results.^36,37^ As shown in Fig. S2(g)–(j),† the O 1s peaks of NiO, Co_3_O_4_, NC-Hydro, and NC-Cal were deconvoluted into two peaks. The peak present at ∼529 eV was due to the M–O bonding, and the peak at ∼531 eV was due to the presence of surface-adsorbed oxygen.^38,39^

## 4. Electrochemical measurements

**Fig. 4:**
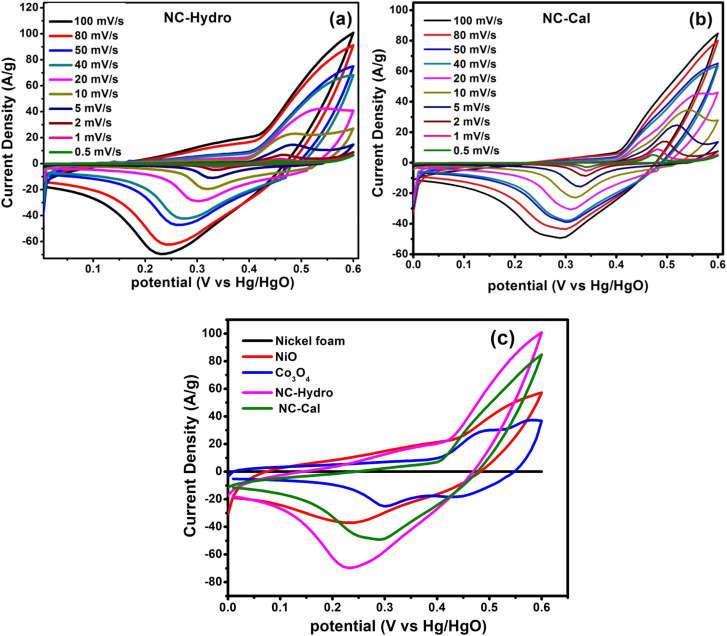
The cyclic voltammetry curves at different scan rates, (a) NC-Hydro, (b) NC-Cal, (c) comparison of cyclic voltammetry among neat nickel foam, NiO, Co_3_O_4_, NC-Hydro, and NC-Cal at a scan rate of 100 mV s^−1^.

In order to investigate the electrochemical properties of NiO, Co_3_O_4_, NC-Hydro, and NC-Cal as a supercapacitor electrode material for supercapacitor applications, cyclic voltammetry (CV) and galvanostatic charge–discharge (GCD) have been studied using a three-electrode electrochemical system with 2 M aqu. KOH solution as an electrolyte. Fig. 4(a) and (b) represent the CV of NC-Hydro and NC-Cal, respectively, with a scan rate ranging from 100 to 0.5 mV s^−1^. Fig. 4(c) displays the CV of NiO, Co_3_O_4_, NC-Hydro, and NC-Cal at 100 mV s^−1^. As depicted in Fig. 4(a) and (b), current increases with the scan rate due to the formation of a thick diffusion layer at lower scan rates, which restricts the electrolyte flux from entering the electrode, resulting in a decrease in current.^40^ The degree of irreversibility and quasi-reversibility increases with an increase in the scan rate, maybe due to the polarization and solution resistance; this results in shifting of oxidation and reduction peaks of NC-Hydro and NC-Cal toward higher and lower potential, respectively.^41^ From the CV patterns of NC-Hydro and NC-Cal, the patterns indicate a battery-like behavior of both the composites. By involving two charge storage processes, the electrochemical charge storage processes occur: (i) the non-faradic and (ii) the faradaic process. These are given below.^19,22,24^

i NiO–Co_3_O_4_ (surface) + OH^−^ ↔ NiO–Co_3_O_4_(OH^−^) surface + e^−^

ii
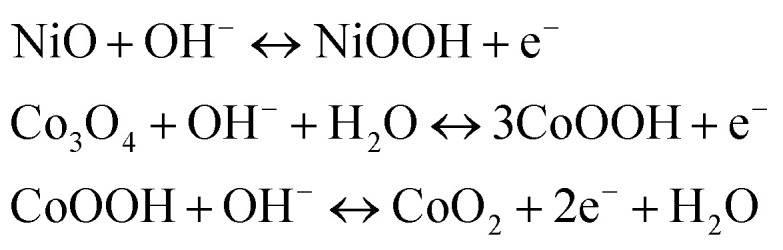


Fig. 4(c) compares the CV currents of neat nickel foam, NC-Cal, NC-Hydro, Co_3_O_4_, and NiO, respectively, at 100 mV s^−1^; the current response of NC-Hydro and NC-Cal is more than that of NiO and Co_3_O_4_, suggesting that both NiO and Co_3_O_4_ have a synergistic effect towards current generation. Furthermore, the current generated by NC-Hydro was more than that of NC-Cal. This may be due to the difference in the synthesis conditions, although the amount of NiO and Co_3_O_4_ used in the synthesis of the composites is the same. NC-Hydro covers a large area when considered along the potential and scan rate axis in comparison to NC-Cal, NiO, and Co_3_O_4_, indicating a large specific capacity of NC-Hydro. The specific capacity of the prepared material is calculated from the CV curve using the following equation.
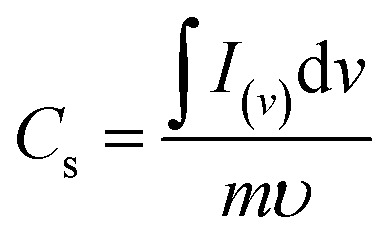
where *C*_s_ is the specific capacity in C g^−1^; ∫*I*_(*v*)_d*v* is the integrated area under the CV curve; *v* is the potential; *υ* is the scan rate in mV s^−1^; and *m* is the mass of the active material in g.^42^ The specific capacity of the NC-Hydro, NC-Cal, NiO, and Co_3_O_4_ at 100 mV s^−1^ is found to be 229, 152, 109, and 56 C g^−1^, respectively. Table 2 represents the specific capacity of NC-Hydro and NC-Cal at different scan rates. From Table 2, it was observed that the specific capacity (C g^−1^) of NC-Hydro is more than that of NC-Cal till a scan rate of up to 40 mV s^−1^; above that, the specific capacity was low in comparison to NC-Cal. The reason behind the high specific capacity of NC-Cal after the scan rate of 40 mV s^−1^ may be due to the high average micro-strain of NC-Cal. At lower scan rates, the defect states are more accessible to the electrolyte, which improves the electrochemical performance.

**Table 2:** The specific capacity of NC-Hydro and NC-Cal at different scan rates

Scan rate (mV s^−1^)		100	80	50	40	20	10	5	2	1	0.5
Specific capacity (C g^−1^)	NC-Hydro	229	241.4	254.2	283.3	359.1	396.2	428	459.6	517.7	707.1
	NC-Cal	151.9	167.6	221.3	258.2	374.6	493.5	586.6	659.4	729.4	864.8

**Fig. 5:**
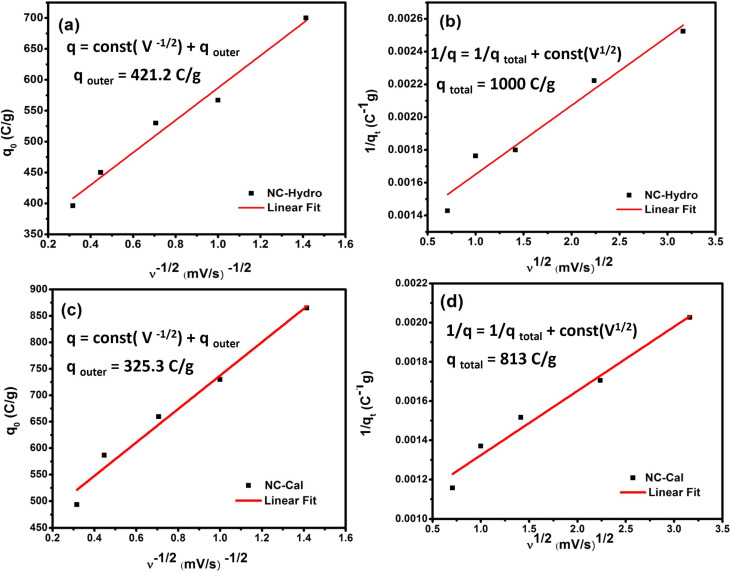
A representation of (a) the plot of *q*_0_ Vs *ν*^−1/2^ and (b) 1/*q* Vs *ν*^1/2^ for NC-Hydro, and (c) the plot of *q*_0_ Vs *ν*^−1/2^ and (d) 1/*q* Vs *ν*^1/2^ for NC-Cal.

**Fig. 6:**
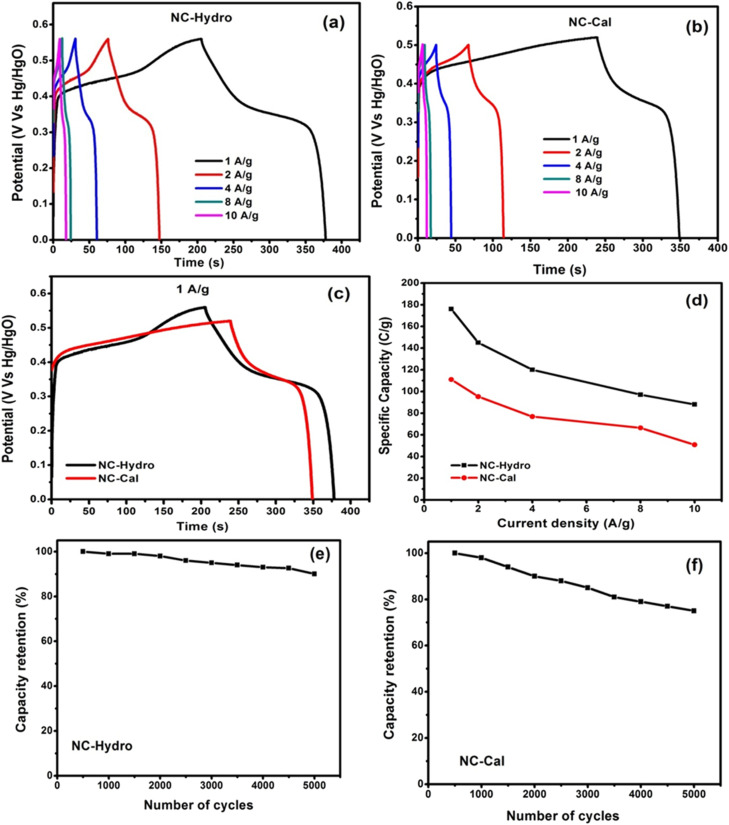
A representation of the GCD curves of (a) NC-Hydro, (b) NC-Cal at different current densities, (c) comparison between GCD curves of NC-Hydro and NC-Cal at a current density of 1 A g^−1^ according to their maximum reachable potential. (d) Specific capacity at various current densities; cyclic retention at a current density of 2 A g^−1^ (e) NC-Hydro and (f) NC-Cal.

The Trasatti method is used to determine the capacitive contribution of surface-controlled charge component (EDLS) and diffusion control charge component (pseudocapacitive) reaction of the electrode materials. The surface-controlled charge component dominates at a high scan rate, while the diffusion-controlled charge component dominates at a low scan rate. Therefore, at a high scan rate, the outer charge (*q*_0_) contributes to capacitance as the access of the electrolyte is restricted only to the outer surface of the electrode material. Contrarily, at low scan rates, the movement of electrolyte is not restricted; it has access to both the inner and the outer surfaces, therefore, the total stored charge can be obtained. The *y*-intercept of the linear fit, *q* Vs *ν*^−1/2^ at *ν* = ∝, shows the outer charge (*q*_0_) stored at the outer surface of the electrode, while the *y*-intercept of the linear fit, 1/*q* Vs *ν*^1/2^ at *ν* = 0, shows the total stored charge (*q*_*t*_). The inner charge (*q*_*i*_) is calculated by subtracting *q*_o_ from *q*_*i*_.^43^Fig. 6 represents the Trasatti plot of NC-Hydro and NC-Cal. Fig. 5(a) and (b) represent the Trasatti plot of NC-Hydro; here, the value of *q*_0_ is found to be 421.2 C g^−1^, and *q*_*t*_ is 1000 C g^−1^. From these values, the value of *q*_*i*_ is calculated to be 578.8 C g^−1^. Similarly, from Fig. 5(c) and (d), the value of *q*_0_ and *q*_*t*_ for NC-Cal is found to be 325.3 and 813 C g^−1^, respectively. The calculated *q*_*i*_ value is 487.7 C g^−1^. In both cases, the value of *q*_*i*_ is found to be greater than that of *q*_0_, suggesting that the inner surface of the electrode possesses a more active surface compared to the outer surface. The result shows the dominancy of the diffusion control charge component (57.8% for NC-Hydro, 60% for NC-Cal) over the capacitive control charge component (42.2% for NC-Hydro, 40% for NC-Cal). The inner charge storage capacity of NC-Cal (60%) is more than NC-Hydro (57.8%), while the outer charge storage capacity of NC-Hydro (42.2%) is more than that of NC-Cal (40%). It is a well-known aspect that the inner charge (*q*_*i*_) arises from the internal regions of voids, grain boundaries, pores, cracks, crevices, *etc.* It is directly linked with micro-strain.^44,45^ The enhancement in the inner charge storage capacity of NC-Cal compared to NC-Hydro may be due to the presence of defect states in the micro-strain region, which improves the conductivity of the active material and increases the number of active sites.^46^ The micro-strain of NC-Cal was found to be more compared to NC-Hydro (as shown in Table 1). This could be the reason behind the greater contribution of NC-Cal towards inner charge storage as compared with NC-Hydro. Furthermore, the electrochemical kinetics of the NC-Hydro was studied by using Dunn's method. The detailed analysis and graphs are included in Fig. S3.†

Further, the charging and discharging capability of NC-Hydro and NC-Cal was studied by galvanostatic charge–discharge curves (GCD curves). The GCD curves of NC-Hydro and NC-Cal are measured at different current densities of 1, 2, 4, 8, and 10 A g^−1^ (Vs Hg/HgO) within the potential range of 0 to 0.56 V (Vs Hg/HgO). For NC-Cal, at 0.56 V, the discharge does not happen, and a parallel line with respect to the time axis is obtained; therefore, 0.5 V is chosen for NC-Cal to study charge–discharge characteristics. Fig. 6(a) and (b) represent the GCD curves of NC-Hydro and NC-Cal at different current densities. The non-linear (voltage plateaus) GCD curves of NC-Hydro and NC-Cal are well-matched with their respective CV curves, indicating their battery-type nature.^47^ The specific capacity was calculated from the GCD curve using the formula, *C*_s_ = *I* × *t*/*m*, where *C*_s_ is the specific capacity at constant current; *I* is the discharge current; Δ*t* is the discharge time; and *m* is the mass of electrode material.^42,43^ The specific capacity of NC-Hydro is found to be 176, 145, 120, 98, and 88 C g^−1^, respectively, for 1, 2, 4, 8, and 10 A g^−1^. The specific capacity decreases with an increase in current density because of the decrease in the diffusion rate of OH- into the electrode surface. Similarly, the specific capacity of NC-Cal is found to be 111, 95.2, 76.8, 66.4, and 50.86 C g^−1^, respectively, for 1, 2, 4, 8, and 10 A g^−1^. CV and GCD provide valuable information on the charge storage behavior of an electrode material. The specific capacity value of the NC-Hydro and NC-Cal obtained from CV and GCD is different because CV and GCD are two different measurement techniques. The capacitance obtained from CV is scan-rate dependent and represents an average over the potential range. On the other hand, GCD measurements involve the application of a constant current, providing a more localized capacitance value at a specific current density.^48,49^

**Fig. 7:**
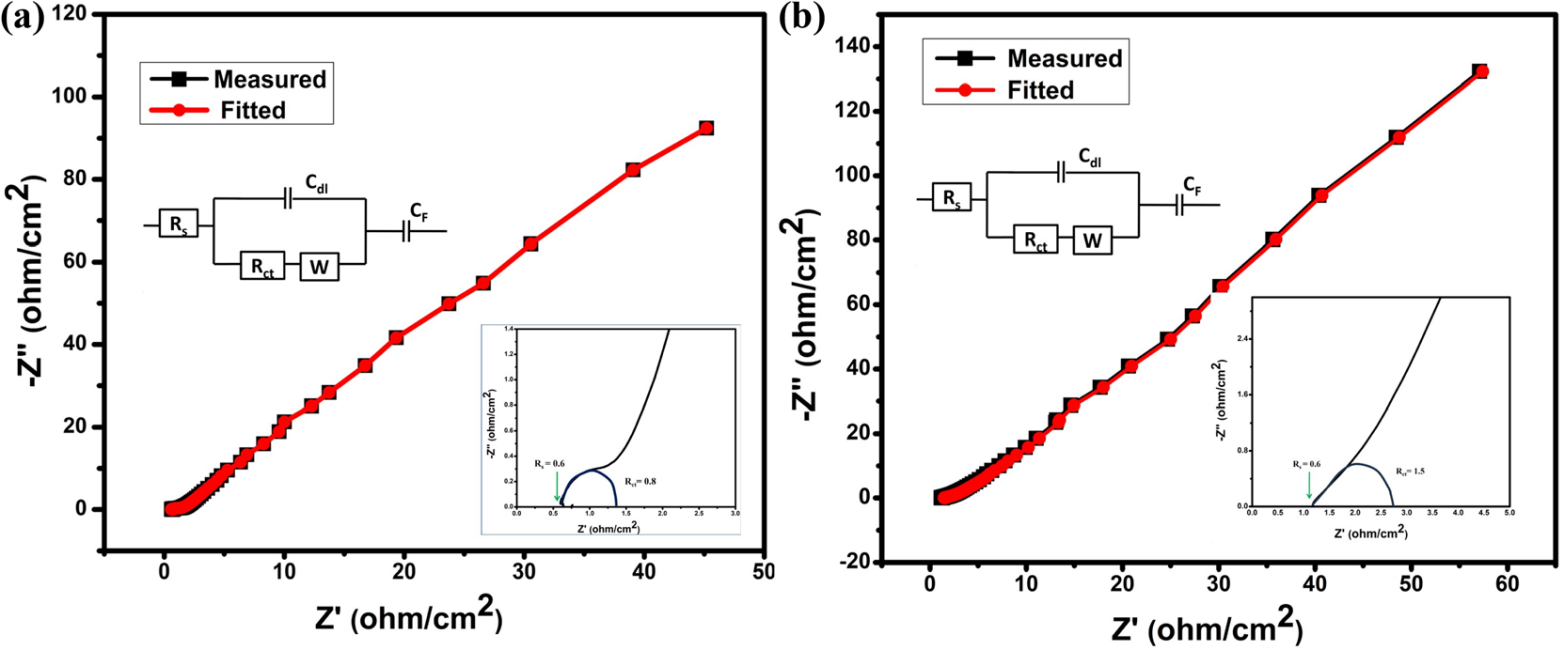
(a and b) Nyquist plot of impedance from 10 mHz to 100 kHz; inset shows equivalent circuit and semi-circle of Nyquist plot for NC-Hydro and NC-Cal.

Fig. 6(c) shows a comparison between GCD curves of NC-Hydro and NC-Cal at a current density of 1 A g^−1^ with respect to their maximum reachable potential. From Fig. 7(c), it is clear that the discharge time of NC-Hydro is more than that of NC-Cal. This may be one of the reasons behind the higher specific capacitance of NC-Hydro than NC-Cal. Fig. 6(d) shows the distribution of specific capacity with respect to current densities. It is found that the NC-Hydro showed ∼55% capacity retention even after the high current density of 10 A g^−1^, while for NC-Cal, it is found to be 46%.

From the coulombic efficiency (*η*) value of any charge storage process, one can easily predict the ease with which the ion insertion or de-insertion may occur. The coulombic efficiency (*η*) of a supercapacitor is represented by the formula, *η* = (Δ*t*D/Δ*t*C) × 100, where Δ*t*C and Δ*t*D are the charging and discharging time, respectively.^41^ By using the formula, the coulombic efficiency of NC-Hydro is found to be 86.3%, which is more than that of NC-Cal (42.3%). Fig. 6(e) and (f) show the cyclic retention of NC-Hydro and NC-Cal, respectively. Approximately, 90 and 75% of the initial specific capacity was retained by NC-Hydro and NC-Cal after 5000 cycles at 4 A g^−1^. This suggests the good rate capability of NC-Hydro in comparison to NC-Cal. FESEM images of NC-Hydro and NC-Cal after the stability study are included in the ESI (Fig. S4†). The reproducibility of the electrode material is an important parameter to test its practical application and commercial viability. To study the reproducibility of NC-Hydro, three working electrodes of NC-Hydro are prepared in nickel foam and named electrode 1, electrode 2, and electrode 3. Reproducibility tests are conducted with GCD measurements in 2 M KOH electrolyte at 2 A g^−1^. The GCD curves of the 3 electrodes exhibit similar patterns (as well as similar specific capacitance) at 2 A g^−1^ as shown in Fig. S5.† In addition to the reproducibility test, the effect of electrolyte, effect of electrolyte concentration, effect of active material ratio, and effect of potential window (w.r.t. Ag/AgCl) have been studied, and the results are shown in Fig. S6.†

From electrochemical impedance spectroscopy, the frequency-dependent super-capacitive behavior of NC-Hydro and NC-Cal is studied in the frequency range of 10 mHz to 100 kHz with a signal amplitude of 5 mV. The EIS data is analyzed by the Nyquist plot. Fig. 7(a) and (b) shows the Nyquist plot of NC-Hydro and NC-Cal; the variables are plotted with an imaginary component (−*Z*′′) *vs.* a real component (−*Z*′) of the impedance; their corresponding fitted equivalent circuit is shown in the inset. The equivalent circuit consists of internal resistance (*R*_s_), charge transfer resistance (*R*_ct_), Warberg (W) impedance, double-layer capacitance (*C*_dl_), and pseudo-capacitance (*C*_p_). The Nyquist plot of NC-Hydro and NC-Cal consists of three regions: (i) high-frequency semicircle, (ii) middle-frequency Warburg impedance, and (iii) low-frequency capacitive behavior. At the high-frequency region, the intercept of the Nyquist plot on the *x*-axis gives the value of *R*_s_. The value of *R*_s_ for the NC-Hydro and NC-Cal is found to be 0.6 and 1.2 Ω, respectively, as shown in the inset of Fig. 7(a) and (b). The *R*_s_ value in the case of NC-Hydro is found to be lower than NC-Cal. The diameter of the semicircle gives the value of *R*_ct_, *i.e.*, 0.8 and 1.5 Ω, respectively, for NC-Hydro and NC-Cal. The *R*_ct_ value of NC-Hydro is found to be lower than NC-Cal, indicating the good conductivity of NC-Hydro.^42,47^ Therefore, it would not be wrong to state that the composite prepared by the hydrothermal method is more suitable for electrochemical reactions rather than by the calcination method. Table 3 presents a comparison of the supercapacitor activity of previously reported NiO/Co_3_O_4_ and our product.

**Table 3:** Comparison of NiO/Co_3_O_4_ materials reported for supercapacitors

Electrode material	Specific capacitance (C g^−1^ or F g^−1^)	Electrolyte	Cyclic stability		Ref.
			Retention (%)	Cycles	
NiO/Co_3_O_4_ core/shell nanofibers	437 F g^−1^ at 1 A g^−1^	6 M KOH	82.9	1000	19
NiO/Co_3_O_4_ nano-heterostructure	405 F g^−1^ at 1 A g^−1^	6 M KOH	97.9	1000	20
NiO/Co_3_O_4_ composite	801 F g^−1^ at 1 A g^−1^	3 M KOH	85	1000	21
Co_3_O_4_/NiO nano films	572 F g^−1^ at 1 A g^−1^	6 M KOH	—	—	22
Co_3_O_4_/NiO nanocomposites	256 F g^−1^ at 10 mV s^−1^	1 M KOH	—	—	23
	233 F g^−1^ at 20 mV s^−1^				
Porous Co_3_O_4_@NiO core–shell	692.8 F g^−1^ at 1 A g^−1^	6 M KOH	90.83	3000	24
This work	541.9 F g^−1^ at 1 A g^−1^ (176 C g^−1^)	2 M KOH	90	5000	
NC-Hydro	660.3 F g^−1^ at 10 mV s^−1^				
	598.5 F g^−1^ at 20 mV s^−1^				
This work	480 F g^−1^ at 1 A g^−1^ (111 C g^−1^)	2 M KOH	75	5000	
NC-Cal	823 F g^−1^ at 10 mV s^−1^				
	624 F g^−1^ at 20 mV s^−1^				

## 5. Conclusions

In summary, forming a heterostructure by combining individual NiO and Co_3_O_4_ is the most favorable approach to improve electrochemical performance. NiO/Co_3_O_4_ composite was synthesized here by adopting two methods: (i) hydrothermal and (ii) calcination. The specific capacity was calculated both from CV and GCD curves. The electrochemical kinetics of the systems were found out from the Trasatti plot. The specific capacity of NC-Hydro and NC-Cal calculated from GCD curves was found to be 176 C g^−1^ and 111 C g^−1^, respectively, at 1 A g^−1^. NC-Hydro showed ∼55% capacity retention at a high current density of 10 A g^−1^, and for NC-Cal, it was found to be 46%. In this study, the activity of the supercapacitor was successfully correlated with the XRD analysis. By analyzing the electrochemical results of NC-Hydro and NC-Cal, it was concluded that the composite prepared by the hydrothermal method performed well as a supercapacitor compared to the composite prepared by the calcination method. This could be due to its large interlayer spacing and lattice parameters that provide a large space for redox reactions to take place. This work may provide new insights into the electrochemical characteristics of the composite with the help of X-ray diffraction results.

## Conflicts of interest

The authors declare no conflicts of interest.

## Supplementary Material

RA-014-D3RA05200A-s001
