# Supplementary material for: Effect of synthesis methods on the activity of NiO/Co3O4 as an electrode material for supercapacitor: in the light of X-ray diffraction study
Source: RSC Adv. 2024 Jan 2;14(1):233–44. doi: 10.1039/d3ra05200a (PMC10759196; doi:10.1039/d3ra05200a)
Supplement: RA-014-D3RA05200A-s001 [file RA-014-D3RA05200A-s001.pdf]

Electronic Supplementary Material (ESI) for RSC Advances.
This journal is © The Royal Society of Chemistry 2023

Effect of Synthesis Methods on the Activity of NiO/Co_3O_4 as an Electrode Material of Supercapacitor: In the Light of X-ray Diffraction Study

Amtul Nashim, Soumyashree Pany, Kulamani Parida*

Centre for Nano Science and Nano Technology, Institute of Technical Education and Research, Siksha 'O' Anusandhan (Deemed to be University), Bhubaneswar, 751019, India,

*Corresponding author

E-mail: paridakulamani@yahoo.com

& kulamaniparida@soa.ac.in

Fig. S1 represented the FESEM images of NC-Hydro and NC-Cal at various magnitudes.

Fig. S2 (a-j) represents the deconvoluted peaks of Ni, Co and O respectively for NiO, NC-Hydro, NC-Cal and Co_3O_4.

Fig. S3 (a) Plots of log (i) versus log (υ) for NC-Hydro. (b) Capacitive and diffusion-controlled process at various scan rate for NC-Hydro.

Fig. S4 FESEM images of (a) NC-Hydro and (b) NC-Cal after the stability study.

Fig. S5 Reproducibility test of the NC-Hydro by taking three different electrodes.

Fig.S6 Effect of different electrolytes on specific capacitance at 10 mVs^{-1}(A) Effect of different molar concentrations of KOH solution over specific capacitance (B) Effect of Mass loading on specific capacitance(C) Effect of potential window on specific capacitance in comparison to Ag/AgCl(D).

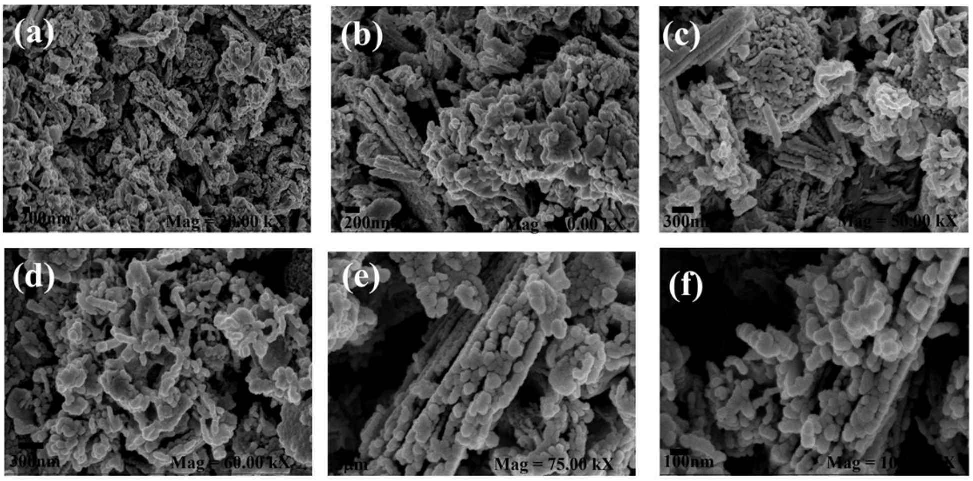

Fig. S1 represented the FESEM images of NC-Hydro at various magnitudes.

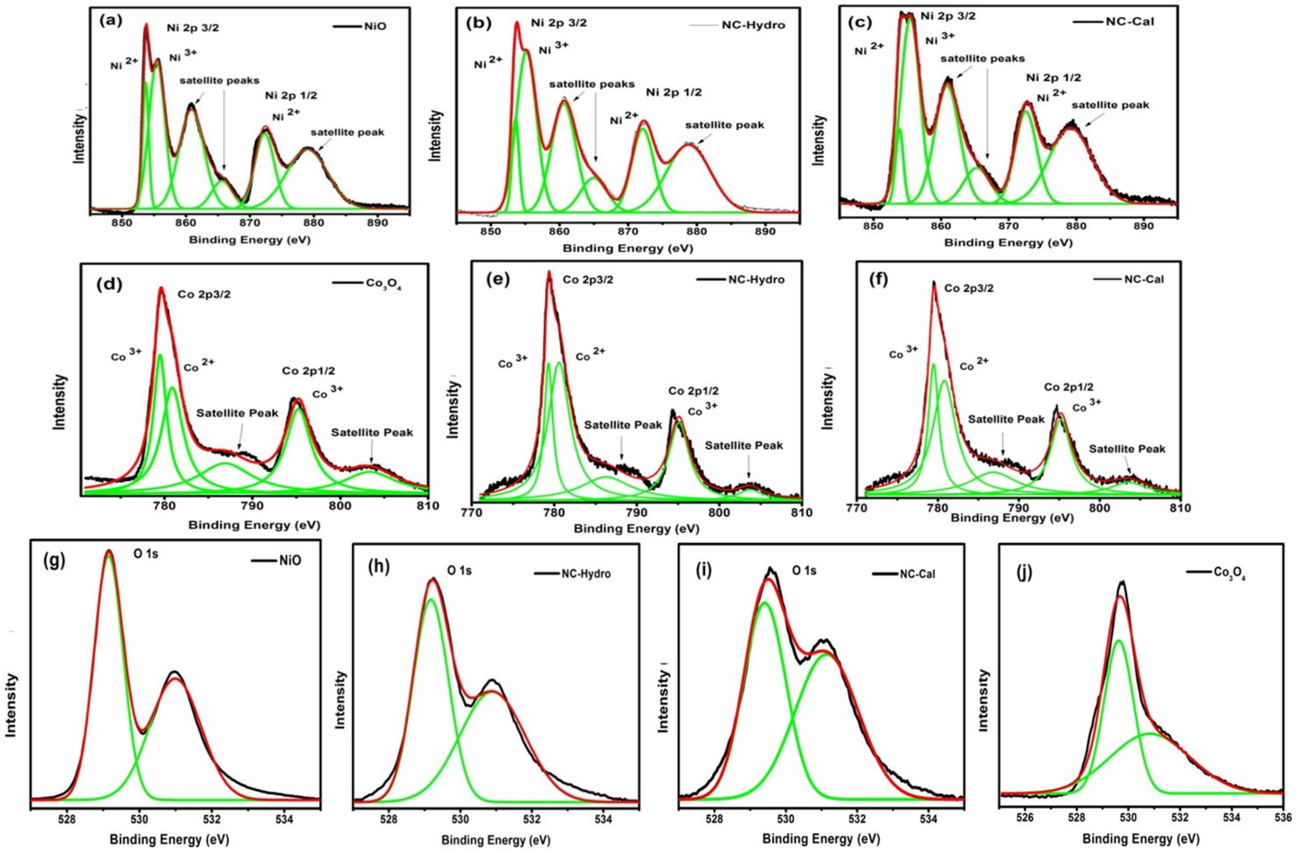

Fig. S2 (a-j) represents the deconvoluted peaks of Ni, Co and O respectively for NiO, NC-Hydro, NC-Cal and Co_3O_4.

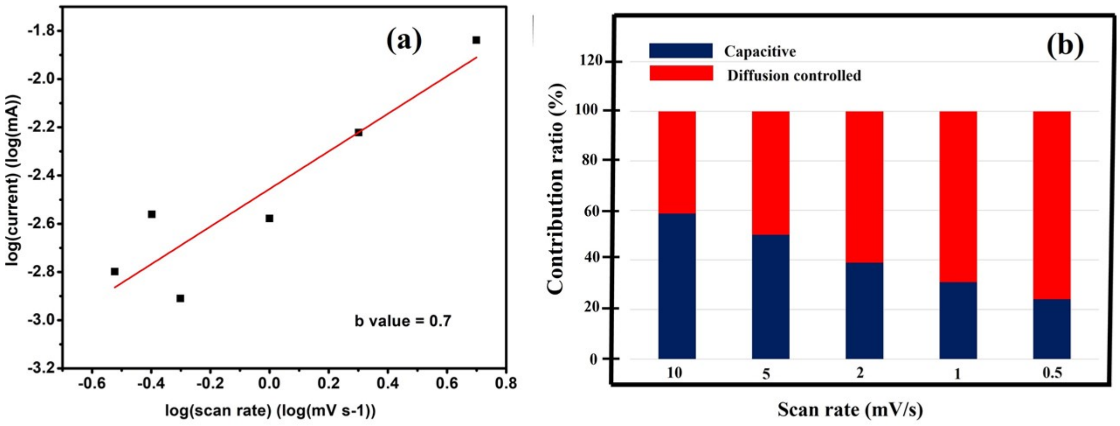

Fig S3 (a) Plots of log (i) versus log (υ) for NC-Hydro. (b) Capacitive and diffusion-controlled process at various scan rate for NC-Hydro.

To distinguish between the capacitive and diffusion-controlled charge storage process; the relationship between scan rate (υ) and peak current (i) is used; and is expressed by power law i.e. $i=\alpha \upsilon^b$ where α and b are constants and b value is determined from the slope of the plot

between log (i) Vs log (υ).[1] For capacitive material b value is 1 and for typical battery type it is 0.5. The transition area between capacitive and typical battery type is represented in the range of 0.5 to 1. Fig S3 (a) represents the calculated b value for NC-Hydro. In this case, the value of b is found to be 0.7. In addition to this the two processes i.e. capacitive controlled process and diffusion controlled process can be estimated by Dunn's method using equation $I(\upsilon)=K_1(\upsilon) + K_2\upsilon^{1/2}$; where $I(\upsilon)$ is the total current at fixed potential, $K_1(\upsilon)$ is the contribution of surface capacitive process and $K_2\upsilon^{1/2}$ is the contribution of diffusion controlled processes.[1] Fig. S3 (b) represents the percentage of capacitive and diffusion-controlled process at various scan rate i.e. 10, 5, 2, 1, 0.5 for NC-Hydro. From Fig S3 (b) it is found that with decrease in scan rate the diffusion-controlled process is predominated.

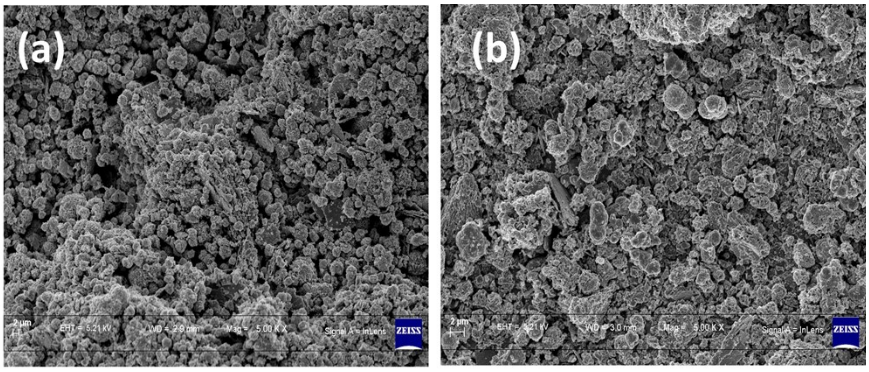

Fig. S4 FESEM images of (a) NC-Hydro and (b) NC-Cal after the stability study.

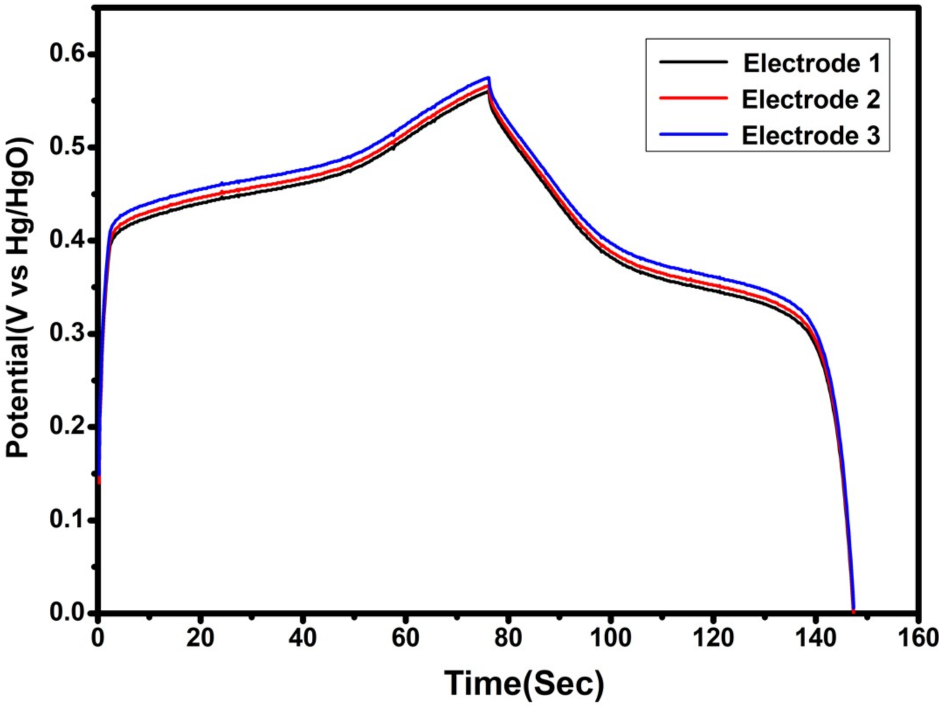

Fig. S5 Reproducibility test of the NC-Hydro by taking three different electrodes.

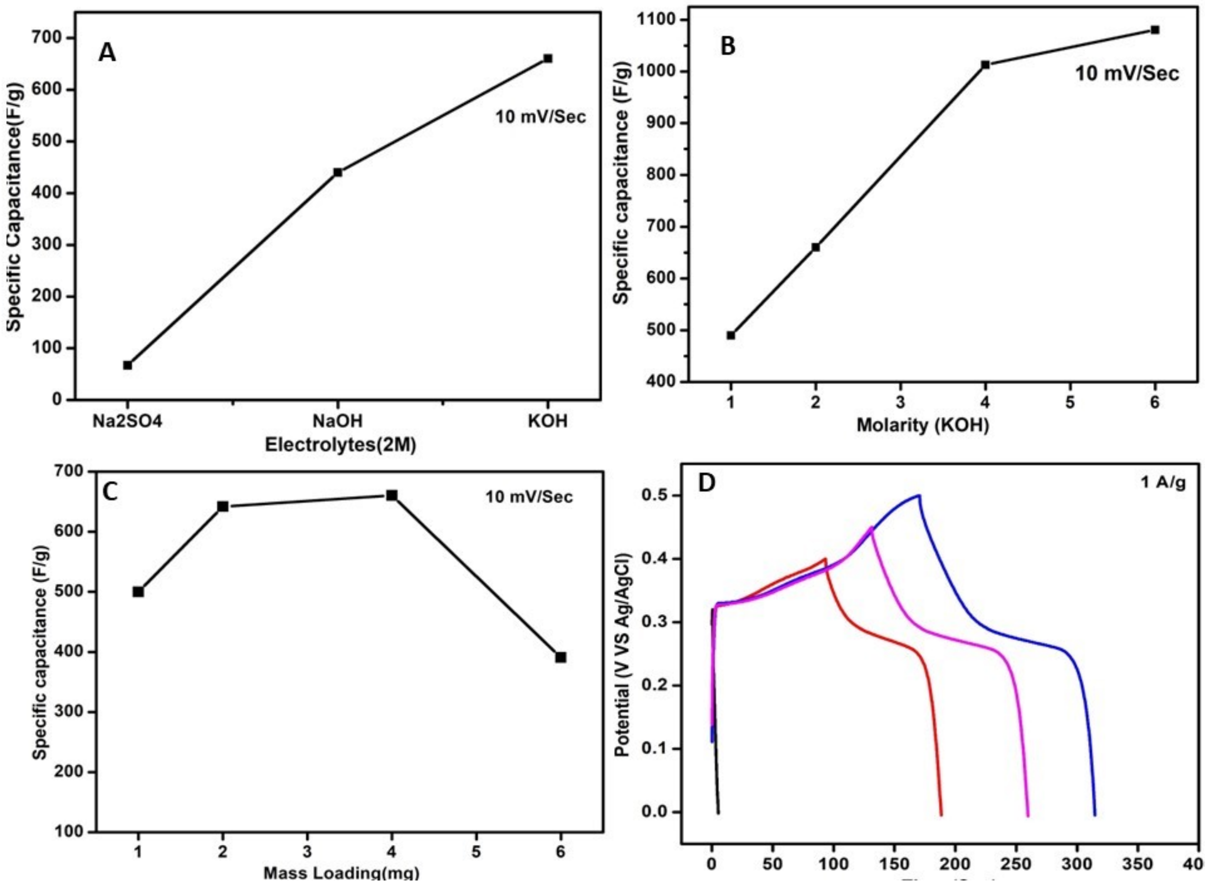

Fig.S6 Effect of different electrolytes on specific capacitance of NC-Hydro at 10 mVs^{-1}(A) Effect of different molar concentrations of KOH solution over specific capacitance (B) Effect of Mass loading on specific capacitance(C) Effect of potential window on specific capacitance in comparison to Ag/AgCl(D).

A detailed comprehensive analysis for NC-hydro (effect of electrolytes, effect of molar concentration, effect of mass loading and effect of potential window) and its effect towards specific capacitance has been performed and is shown in Fig.S6. The specific capacitance of NC-hydro with different electrolyte has been studied and is shown in Fig. S6 (A). The specific capacitance observed for NC-hydro is 660.3 F/g at 10 mV/sec in 2M KOH. The capacitance performance with different electrolyte decreases in the order 2MKOH > 2M NaOH> 2M Na_2SO_4. The above trend might be due to the differences in ionic radius of the electrolytes, the radius of ionic hydration sphere of electrolytes, the conductivity of the ions and the mobility of the ions. [2,3] Fig. S6 (B) represents effect of different molar concentration of KOH solution over specific capacitance of NC-hydro. Here, from the observation, we noticed gradual increase

in specific capacitance of NC-hydro as the concentration of KOH varies from 1M to 6M. Generally, the conductivity of an electrolyte increases with its concentration in aqueous solution. Thus, as the electrolyte concentration increases, it might be providing higher conductivity and more enough OH^- concentration, which facilitates charge transfer in both bulk electrolyte and electrode. [4] Therefore, here 6M KOH offer high specific capacitance performances in comparison to 1M, 2M and 4M KOH. Fig.S6 (C) represents effect of mass loading of NC-hydro on specific capacitance at a scan rate of 10 mV s^{-1}. Here, from the observation, we noticed increase in mass loading of NC-hydro (from 1-4mg) on nickel foam increases specific capacitance values up to 660.3 F/g at 10 mV s^{-1}. Further deposition (6mg) of NC-hydro on nickel foam results decrease in specific capacitance. This phenomenon at higher mass loading might blocked electron and ion transport channels and reduces active sites as a result specific capacitance value is decreases. [5] Fig.S6 (D) represents effect of potential window on specific capacitance in comparison to Ag/AgCl electrode. Here, from the observation NC-hydro in 2M KOH shows a limited potential window (i-e 0-0.5) as well as unstable GCD profile nature for Ag/AgCl electrode in comparison to Hg/Hgo electrode. This might limit the specific capacitance performance as it restricts charge storage capacity and stability performance in a wider potential window.

References:

1. Y. Lan, H. Zhao, Y. Zong, X. Li, Y. Sun, J. Feng, Y. Wang, X. Zheng, Y. Du. *Nanoscale,* 2013*,* **25**,11775-11781.
2. S. Chaudhari, D. Bhattacharjya, J.S Yu, *RSC Adv.*, 2013, **3**, 25120-25128.
3. V.D. Nithya, R. K. Selvan, D. Kalpana, L. Vasylechko, C. Sanjeeviraja, *Electrochim. Acta*, 2013,**109**, 720-731.
4. Y. Zhao, M. Hao, Y.Wang, *J Solid State Electrochem* ,2016, **20**, 81-85.
5. Y. Dong, J. Zhu, Q. Li, S. Zhang, H. Song, J. Dianzeng, *J. Mater. Chem. A*, 2020, **8**, 21930-21946.
